# Clinical significance of troponin elevations in acute decompensated diabetes without clinical acute coronary syndrome

**DOI:** 10.1186/1475-2840-11-154

**Published:** 2012-12-27

**Authors:** Anthony Eubanks, Farhan Raza, Mohamad Alkhouli, April N Glenn, Carol Homko, Abul Kashem, Alfred Bove

**Affiliations:** 1 Cardiology Section, Temple University School of Medicine, Philadelphia, USA; 2 Department of Internal Medicine, Temple University School of Medicine, Philadelphia, USA; 3 Temple University Medical Center – Cardiology Section, 3401 North Broad Street, Room, Philadelphia, PA, 19140, USA

**Keywords:** Cardiac troponin-I, Decompensated diabetes, Prognostic markers, Acute coronary syndrome, CK-MB

## Abstract

**Background:**

Elevation of cardiac troponin has been documented in multiple settings without acute coronary syndrome. However, its impact on long-term cardiac outcomes in the context of acute decompensated diabetes remains to be explored.

**Methods:**

We performed a retrospective analysis of 872 patients admitted to Temple University Hospital from 2004–2009 with DKA or HHS. Patients were included if they had cardiac troponin I (cTnI) measured within 24 hours of hospital admission, had no evidence of acute coronary syndrome and had a follow up period of at least 18 months. Of the 264 patients who met the criteria, we reviewed the baseline patient characteristics, admission labs, EKGs and major adverse cardiovascular events during the follow up period. Patients were categorized into two groups with normal and elevated levels of cardiac enzymes. The composite end point of the study was the occurrence of a major cardiovascular event (MACE) during the follow up period and was compared between the two groups.

**Results:**

Of 264 patients, 24 patients were found to have elevated cTnI. Compared to patients with normal cardiac enzymes, there was a significant increase in incidence of MACE in patients with elevated cTnI. In a regression analysis, which included prior history of CAD, HTN and ESRD, the only variable that independently predicted MACE was an elevation in cTnI (p = 0.044). Patients with elevated CK-MB had increased lengths of hospitalization compared to the other group (p < 0.001).

**Conclusions:**

Elevated cardiac troponin I in patients admitted with decompensated diabetes and without evidence of acute coronary syndrome, strongly correlate with a later major cardiovascular event. Thus, elevated troponin I during metabolic abnormalities identify a group of patients at an increased risk for poor long-term outcomes. Whether these patients may benefit from early detection, risk stratification and preventive interventions remains to be investigated.

## Background

Elevated cardiac biomarkers in decompensated diabetes in the absence of an acute coronary syndrome (ACS) have been described in several case reports [1-5]. While non-ACS related cardiac biomarkers have been studied in various acute and chronic medical conditions, acute decompensated diabetes has received less attention [1,5-12]. Acute decompensated diabetes and ACS, share a complex dynamic that results in significant ambiguity when interpreting biomarker elevation in this setting [13-15]. Such ambiguity is concerning because myocardial infarction has been shown to be the most common cause of death within the first 24 hours of admission for acutely decompensated diabetes [16].

Recent studies have highlighted a novel relationship between the severity of acidemia in acute decom-pensated diabetes and abnormal elevations in cardiac troponin-I (cTnI). Moller et al. describe patients in diabetic ketoacidosis with severe acidemia and abnormally elevated cTnI who had no angiographic evidence of coronary artery disease (CAD), leading them to suggest that ketoacidemia may contribute to elevations in cardiac enzymes [4].

Since the number of hospital discharges for acute decompensated diabetes has doubled since 1980 [17] and the worldwide incidence of diabetes mellitus (DM) is expected to double over the next 15 years [18-22], defining the importance of elevated cardiac biomarkers in diabetic disorders is critical. In this study, we assessed the clinical significance of abnormal elevations in cTnI in decompensated diabetics.

## Methods

We performed a retrospective review of 872 charts for patients admitted to Temple University Hospital (TUH) with a diagnosis of “Diabetic Ketoacidemia” between 2004–2009. Approval for this chart review was obtained from the Institutional Review Board. Inclusion criteria required patients to have levels of cTnI within 24 hours of admission, a history of prior or newly diagnosed diabetes mellitus, and evidence of diabetic ketoacidemia (DKA) or the hyperosmolar hyperglycemic state (HHS). DKA and HHS were defined in accord with common clinical practice [14,15]. 298 patients met inclusion criteria.

For patients that met inclusion criteria, if levels of CK-MB were also measured, serum values for both biomarker sub-types were recorded. Patients were considered to have abnormal elevations in cardiac biomarkers if either CK-MB (0.00-7.50 ng/ml) or cTnI (0.05-0.40 ng/ml) were above the hospital’s normal reference level.

**Figure 1:**
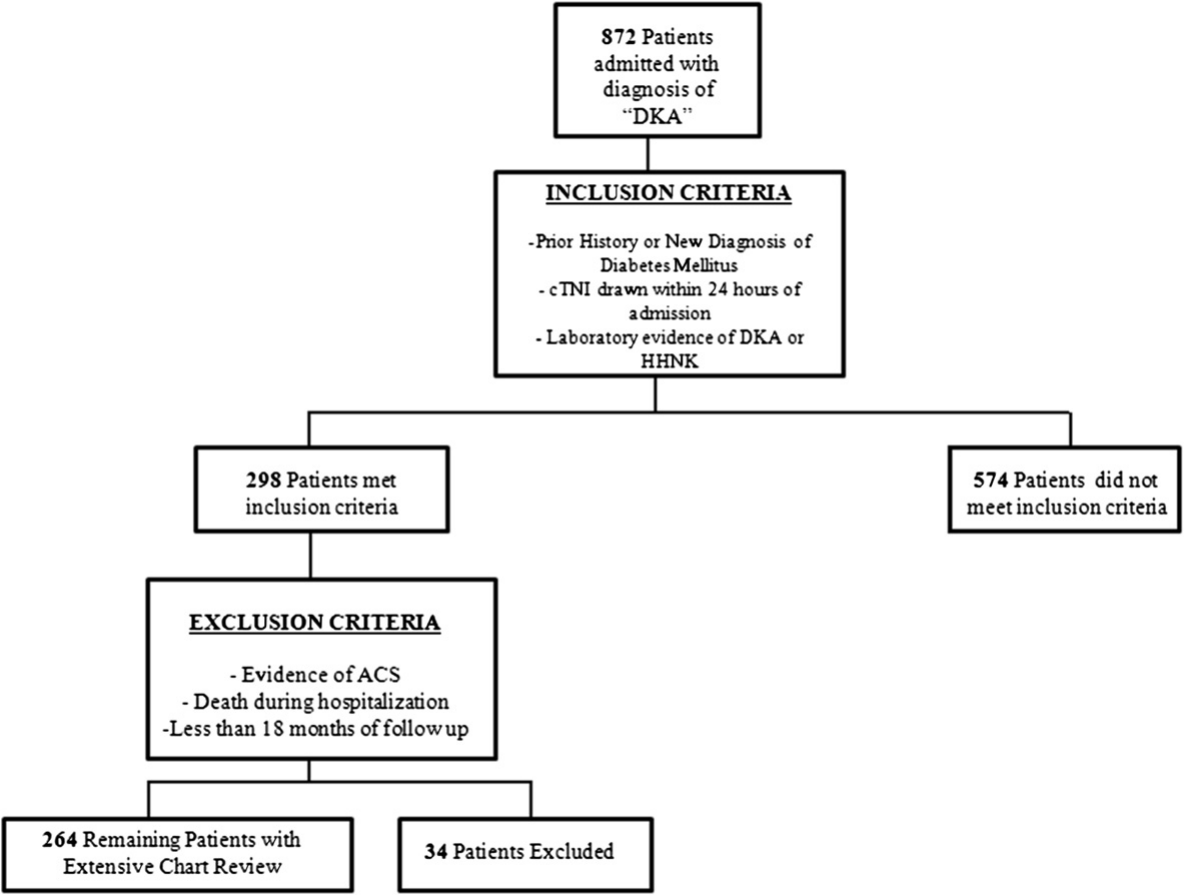
**Flow Chart Identifying Patient Selection.** Abbreviations: ACS = Acute Coronary Syndrome; DKA = Diabetic Ketoacidosis; HHNK = Hyperglycemic Hyperosmolar Non-Ketotic Syndrome.

Patients were excluded from the study if they had evidence of acute coronary syndrome in accord with the AHA/ACC Guidelines [18], or if the patient died during the hospitalization. ECGs were analyzed retrospectively by two separate physicians blinded to clinical outcomes. An ECG was determined to be consistent with ACS if there was ≥1 mm ST depression or ≥2 mm ischemic T wave inversion, new Q- waves, new left bundle branch block, or ST elevations consistent with ischemia. Of the 298 patients who met inclusion criteria, 34 were excluded because of suspected or confirmed ACS, or death during admission (See Figure 1). For the remaining 264 patients, prior medical history, and key hospital data were recorded. Using the electronic medical record, Alpha Imaging Systems®, Centricity®, PACS® image viewer and EPIC®, both inpatient and outpatient medical records were reviewed to record baseline patient characteristics, length of hospital stay, length of follow up, and to assess the time interval in months to the occurrence of a major adverse cardiac event (MACE). MACE was defined as myocardial infarction, percutaneous coronary intervention (PCI), coronary artery bypass grafting (CABG), or death. Only the first admission was included for patients admitted more than once. chronic renal insufficiency was defined as baseline creatinine above 1.4 mg/dL, end stage renal disease (ESRD) was defined as patients on hemodialysis or peritoneal dialysis.

### Statistical analysis

Continuous variables were expressed as mean ± SE and statistical significance was tested using the Student t- test. Categorical variables were analyzed using either the chi-square statistic or Fischer exact test, as appropriate. Patients were grouped as normal versus elevated based upon serum admission cTnI levels. Multivariate logistic regression models were constructed to determine variables that predicted abnormal elevations in admission serum cTnI. Similar models were constructed to identify variables that independently predicted long term outcomes. Linear regression models were constructed to identify variables that predicted length of hospital stay. Length of hospital stay and MACE were chosen as objective measures for short and long term outcomes respectively. Kaplan-Meir plots were used to assess effects over time. Associations were considered significant if alpha < 0.05. All analyses were performed with SPSS. v19.0

### Measurement of cardiac troponin I

The blood specimens analyzed in this study were collected in observation with routine precautions for venipuncture. Blood samples were allowed to clot completely prior to centrifugation and stored at room temperature (15 to 30°C) for no longer than two hours.

Cardiac troponin I was measured by the Access AccuTni™ chemiluminescent immunoassay (Beckman Coulter®). This assay uses two monoclonal antibodies in conjunction with alkaline phosphatase to bind antigenic sites in the solid phase, resulting in a complex between human cTnI and monoclonal anti-cTnI antibody. The Access AccuTni™ chemiluminescent immunoassay has a cTnI cutoff of 0.4ng/mL, this cutoff yields the most optimal sensitivity and specificity.

### Measurement of CK-MB

Similar to cTnI, CK-MB was also measured with the Access AccuTni™ chemiluminescent immunoassay, with a similar two monoclonal antibody system.

## Results

**Figure 2:**
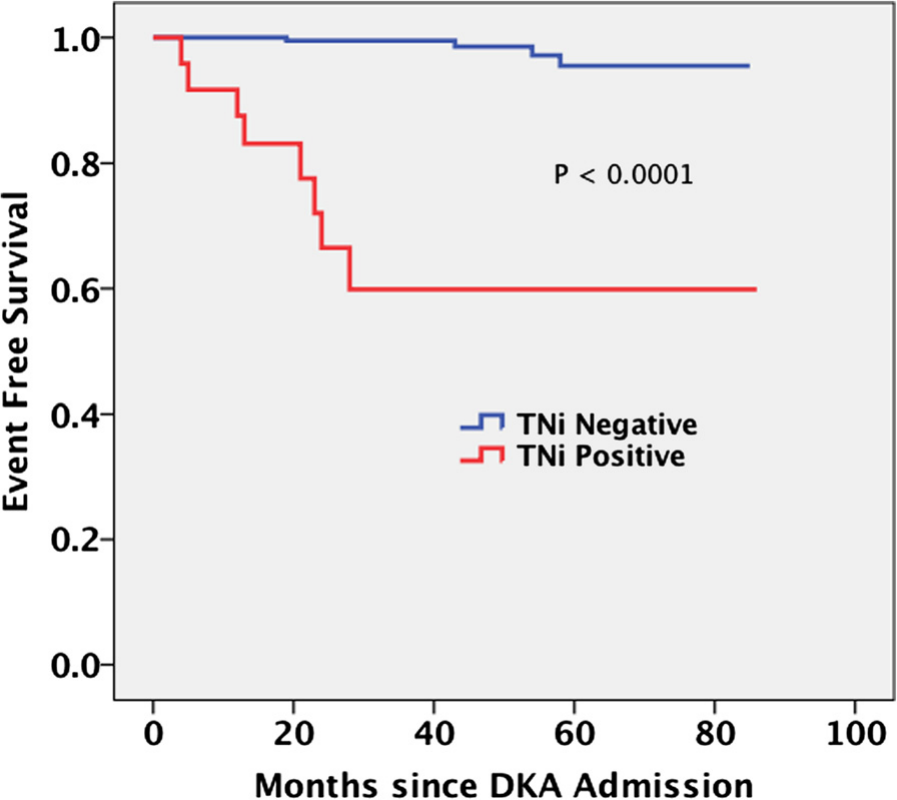
**Survival Function in Months Between Based Upon Serum Admission Biomarkers.** Kaplan-Meier plot reveals statistically significant survival in both the negative troponin group compared to the positive troponin group (with maximum difference over first two years).

Demographic characteristics of the patient population based upon serum admission cTnI and their clinical measures at admission are shown in Tables 1 and 2. Of 264 patients, 24 patients were found to have elevated cTnI. There were no statistically significant differences in age, sex or ethnic makeup identified amongst the studies participants when comparison was made based upon patients with normal versus elevated admission cTnI. Subjects with elevated admission serum cTnI had a greater prevalence of prior coronary artery disease, hypertension and chronic renal insufficiency when compared to their counterparts with normal levels. Blood glucose and serum admission pH were inversely proportional as seen in Figure 2.

**Table 1:** Descriptive statistics – continuous data

**Variable**	**Normal cTnI**	**Elevated cTnI**	**P value**
Age(Years)	45.1 ± .88	50.3 ± 3.41	.081
Blood			
Glucose (mg/dL)	690.0 ± 17.30	1045 ± 82.30	<0.001
Hemoglobin (gm/dL)	14.30 ± 0.27	12.50 ± 0.60	0.040
pH	7.18 ± 0.01	7.07 ± 0.04	<0.001
Anion Gap (mmol/L)	26.00 ± 0.42	28.13 ± 1.67	0.138
Creatinine (mg/dL)	2.40 ± 0.12	4.22 ± 0.58	0.004
Mean length of Follow Up (Months)	41.6 ± 1.31	37.9 ± 4.10	0.691

**Table 2:** Descriptive statistics – categorical data

**Variable**	**Normal cTnI**	**Elevated cTnI**	**P value**
%Male	50.0	50.0	.479
White (%)	20.5	20.8	.942
Black (%)	69.0	62.5	.942
Latino (%)	7.9	8.3	.942
Other (%)	2.5	4.2	.942
History of CAD (%)	4.6	41.7	<0.001
CRI (%)	9.7	12.5	0.66
ESRD (%)	2.1	8.3	0.07
Tobacco (%)	53.2	45.0	0.48
HTN (%)	45.6	69.6	0.028

Abbreviations: CAD = Coronary Artery Disease; CRI = Chronic Renal Insufficiency; ESRD = End Stage Renal Disease; HTN = Hypertension.

Subjects with elevated serum cTnI had a significantly higher incidence of post discharge MACE when compared to normal subjects (Table 3). The increase resulted from a high incidence of myocardial infarction post discharge. Additionally, hospital length of stay was higher (Table 3) in the subjects with elevated admission serum cTnI when compared to their normal counterparts.

**Table 3:** Study outcomes amongst subjects enrolled based upon serum admission cTnI

**Variable**	**Normal cTnI**	**Elevated cTnI**	**P value**
MACE (%)	1.70	33.00	<0.001
MI (%)	1.70	33.00	<0.001
CABG (%)	0.00	0.00	-
PCI (%)	0.42	0.00	<0.001
Death (%)	0.00	4.20	<0.001
Mean Length of Hospital Stay (Days)	5.73 ± 0.37	11.04 ± 1.97	<0.001

Abbreviations: CAD = Coronary Artery Disease; CRI = Chronic Renal Insufficiency; ESRD = End Stage Renal Disease; HTN = Hypertension.

### Factors that predict elevated cTnI

Table 4 shows the factors that made significant contributions to cTnI elevation. A history of coronary disease, low pH (less than 7.1), and elevated CK-MB levels were independent predictors for an elevated cTnI level.

**Table 4:** Multi-variable logistic regression for predicting elevations in cTnI

**Variable**	**P value**	**O.R.**	**95% C.I. low**	**95% C.I. high**
Age	0.484	0.984	0.940	1.030
CAD	<0.0001	15.9	3.7	68.7
pH	0.027	0.047	0.003	0.706
HTN	0.419	1.70	0.471	6.10
Gender	0.441	0.65	0.21	1.96
Chronic Renal Insufficiency	0.680	0.71	0.14	3.70
Elevated Serum CK-MB	<0.0001	11.1	3.3	37.1

Abbreviations: CAD = Coronary Artery Disease; CK-MB = Creatine Kinase – MB.

### Factors that predict MACE

Table 5 shows the contribution of clinical factors to risk for MACE following hospital discharge. Using multivariate logistic regression, a statistically significant relation was only found for an elevated admission serum cTnI, however pH also shows a strong trend toward influencing MACE. The other clinical factors listed in Table 5 do not show significant relationships with MACE. The odds ratio (OR) for pH demonstrates an increased risk of MACE for decreased levels of pH. Independent analysis of pH influence on MACE using univariate logistic regression identified a serum admission pH of less than 7.1 as statistically significant independent predictor for MACE.

**Table 5:** Multi-variable logistic regression for predicting MACE

**Variable**	**P value**	**OR**	**95% C.I. low**	**95% C.I. high**
Prior History of CAD	0.127	3.659	−0.369	2.963
Elevated cTnI	0.044	1.068	0.002	0.129
Hypertension	0.085	5.312	−0.228	3.567
Chronic Renal Insufficiency	0.122	3.510	−0.335	2.846
pH	0.055	0.21	−6.907	0.083
Age	0.630	1.013	−0.039	0.065
Gender	0.395	0.543	−2.017	0.796
Race	0.329	0.422	−2.590	0.567
Elevated CK-MB	0.638	1.004	−0.011	0.018

Abbreviations: CAD = Coronary Artery Disease; CK-MB = Creatine Kinase – MB; Cardiac Troponin-I – cTnI.

**Figure 3:**
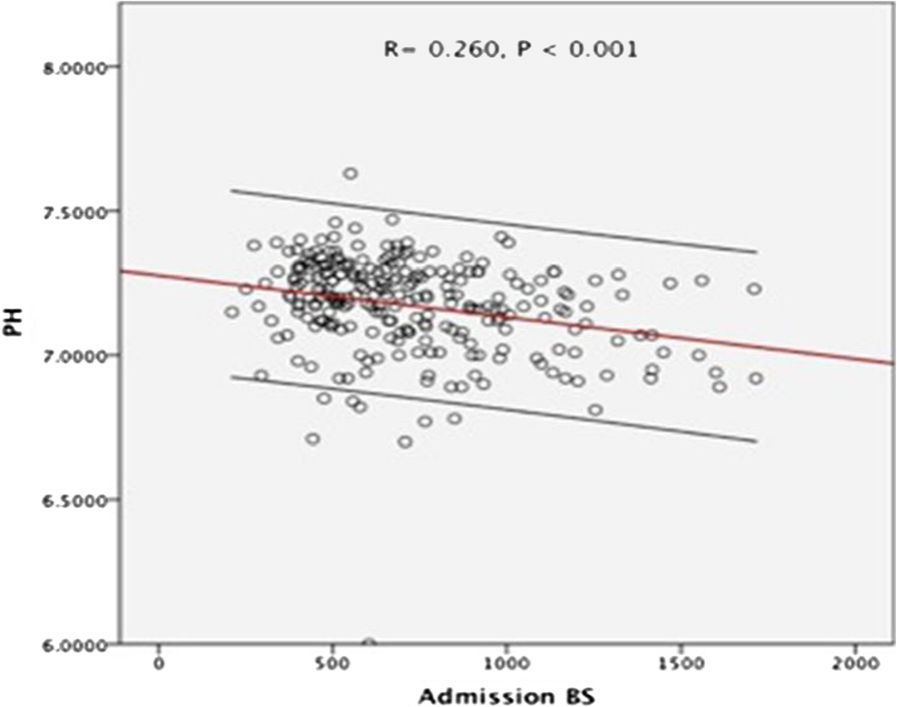
Graphical Representation of Serum Glucose versus Serum pH At Time of Admission.

Figure 3 shows a significant difference in event-free survival from MACE when comparing patients with and without elevated admission serum cTnI. The freedom from MACE is significantly lower in subjects who demonstrated elevated cTnI during hospitalization in the absence of evidence for an acute coronary syndrome.

### Factors that predict length of hospital stay

Patients with an elevated serum cTnI at admission had longer lengths of stay. Between the two biomarkers, only CK-MB was a statistically significant independent predictor for length of stay (Table 6).

**Table 6:** Multi-variable linear regression for predicting length of hospital stay

**Variables Considered**	**B Coefficient**	**95% C.I. low**	**95% C.I. high**	**P value**
Age	0.071	0.023	0.120	.004
Gender	−0.443	−1.755	.868	.506
Chronic Renal Insufficiency	0.686	−1.643	3.016	.562
pH	−2.363	−6.359	1.634	0.245
Elevated cTni	−0.001	-.108	0.106	0.986
Elevated CK-MB	0.109	0.082	0.137	0.000
Hypertension	0.466	−0.962	1.893	0.521
Race	−0.613	−1.494	0.268	0.172

Abbreviations: CK-MB = Creatine Kinase – MB; Cardiac Troponin-I – cTnI.

## Discussion

This study identified independent prognostic factors that predict elevations in serum admission cTnI, and demonstrated elevated admission serum CK-MB and cTnI as prognostic for short and long term outcomes respectively. As a result, we gained insight into the clinical significance of elevated admission serum cardiac biomarkers in acute decompensated diabetes in the absence of clinically evident ACS. In the future, these data can be used to guide management.

An admission serum pH of less than 7.1 was demonstrated to be an independent prognostic factor for elevations in serum admission cTnI. To our knowledge, this is the first study to demonstrate a relationship between serum pH and elevated serum troponin. Our study supports the concept put forth by Moller et al., who noted that patients with elevated cTnI did not have angiographic evidence of coronary artery disease but had serum admission pH values of less than 6.9 [4].

What accounts for the correlation between cTnI and serum pH in the context of this study is unclear, and remains to be elucidated. A potential explanation highlights a complex dynamic between pH and intracellular calcium. As a result of severe acidemia, there is an increase in intracellular calcium which activates multiple biochemical pathways including proteolysis and myocardial stunning culminating in increased serum cTnI [23-28].

Approximately 40% of the patients with an elevated serum admission cTnI had a documented prior history of CAD. Regression analysis demonstrated a prior history of CAD as a prognostic factor for elevated cTnI. Acute decompensated diabetes is characterized by increased levels of counter-regulatory hormones which increase myocardial oxygen demand [14-16]. In such a population, the added effect of CAD likely impairs blood flow, exacerbating a supply–demand mismatch, resulting in myonecrosis and elevations in cTnI.

Logistic regression models identified an elevated admission serum cTnI as the only statistically significant variable for the long term composite outcome, MACE. Of interest is the finding that CK-MB was not correlated with the long term MACE outcome. Furthermore, an elevated admission serum cTnI proved to be a better predictor for MACE than a prior history of CAD (p = 0.127). MACE was primarily driven by myocardial infarction, thus it was unexpected that a prior history of CAD was not a statistically significant independent predictor. More likely, MACE incidence represents multiple pathophysiologic mechanisms, independent of a prior history of CAD alone.

One factor, likely playing a major role in this cohort is insulin resistance. Recent literature has recognized more aggressive forms of type 2 diabetes marked by greater insulin resistance [16,20,29-34]. When these patients decompensate, they have increased production of ketoacids and more severe acidemia. Over time, insulin resistance leads to increased levels of pro-inflammatory cytokines like CRP and homocysteine that accelerate atherogenesis, plaque rupture, and MACE. This study noted an admission serum pH of less than 7.1 to trend toward statistical significance in an unadjusted logistic regression, and after adjusting for other variables, demonstrated a statistical significance for MACE. Demographically, patients with such aggressive forms of diabetes mellitus tend to be African American, in major urban cities. In comparison, 70% of the patients in our study were African Americans in a major urban city. The data suggest that patients in our study have a more aggressive form of diabetes mellitus that predisposes them to MACE independent of a prior history of CAD, and thus are at increased risk.

Concomitantly, in the context of non-diabetic patients status-post myocardial infarction, Knudsen et al. advance our premise. In an article published in 2010, they present a preponderance of evidence which highlights the connection between pro-inflammatory cytokines and deranged blood glucose control [35].

Studies by Hernandez et al. noted that 21% of diabetics have silent ischemia, and Zheng et al., suggest patients with diabetes have a chronic level of myocardial injury, and identified a correlation with blood glucose and elevated levels of serum hs-cTnT [36,37]. Populations such as this cohort, which stand at increased risk for adverse cardiac events underscore the need for future studies that further characterize a complex pathway.

Our findings corroborate those of Al-Mallah et al. who identified cTnI as prognostic for long term cardiovascular events in the context of DKA [38]. This study differed in that we demonstrated a different relationship of MACE to Troponin and to CK-MB. Further, our study identified independent prognostic variables that predict elevations in serum cTnI, lending an objective basis to explain their clinical significance. Further, we assessed short term outcomes, and identified CK-MB as an independent predictor for length of hospital stay. Thus, we define a novel role for CK-MB in the context of acute decompensated diabetes. Regarding patient characteristics, while both studies were relatively small, our study had nearly twice as many participants enrolled (n = 264 v n = 96) and our average length of follow up was nearly twice that of the prior study (40 months v 24 months).

### Study limitations

This study was retrospective with a small sample size. In the future there is a need for larger multicenter prospective studies. Additionally, we lacked angiographic evidence at time of admission required to characterize coronary anatomy and thus patients were excluded based upon clinical information alone.

## Conclusions and clinical implications

As a result of this study, we have identified independent prognostic factors which can help guide clinical management and predict elevations in cTnI, as well as short term and long term outcomes. While a prior history of CAD. Disease plays a key pathophysiologic role, the severity of acidemia appears to be equally important. cTnI and CK-MB should be measured in all patients with acute decompensated diabetes, if elevated, in the absence of clinically evident ACS, findings in this study identify a patient population that is at increased risk for a longer hospital stay in the short term, and increased risk for MACE post-discharge.

## Abbreviations

ACS: Acute coronary syndrome; cTnI: Cardiac troponin-I; CAD: Coronary artery disease; DM: Diabetes mellitus; TUH: Temple University Hospital; HHNK: Hyperosmolar hyperglycemic non-ketotic syndrome; MACE: Major adverse cardiac event; ESRD: End stage renal disease; PCI: Percutaneous coronary intervention; CABG: Coronary artery bypass grafting; HTN: Hypertension; MB – CK-MB: Creatine kinase.

## Competing interests

None of the authors have competing interests to disclose.

## Authors’ contributions

AE worked to collect data, research background articles, and draft the initial version of the manuscript. FR worked to collect data, research background articles and provide key editorial input for subsequent drafts of the manuscript. MA initiated the experimental design, researched background articles, interpreted EKGs, provided key editorial input for subsequent drafts of the manuscript. AG worked to edit the collected data, provide key editorial input for subsequent drafts, and played a key role in analyzing the study results and was very active in drafting the initial discussion section. HK provided key insight into the final outcomes, study design, editorial review and final version of the manuscript. AK worked throughout all stages of the study to provide key insight regarding the study outcomes, interpreting data, researching background articles and validation of statistical analysis. AB served as the principal investigator and worked in all stages of the study including statistical analysis, editorial review, and provided key insight regarding interpretation of the data. All contributions from authors involved were essential in the development of the manuscript. All authors read and approved the final manuscript.
